# Treating Borderline personality disorder with Asenapine : Case report

**DOI:** 10.1192/j.eurpsy.2023.2065

**Published:** 2023-07-19

**Authors:** S. K. I. M. Aboelenien, M. Iderapalli

**Affiliations:** 1 CMHT-Southend, Essex Partnership university trust (EPUT), Rochford, United Kingdom

## Abstract

**Introduction:**

Borderline personality disorder (BPD) is a common and serious mental disorder. Its prevalence is estimated to be around 20% among psychiatry inpatients and 11% in Psychiatric Outpatients. Patients with BPD present a wide range of psychopathological symptoms such as affective instability, impulsivity, interpersonal problems, psychotic-like symptoms and self-destructive behaviour. BPD also occurs as comorbid illness with number of other Psychiatric diseases. There is no psychotropic medication that has been approved by FDA or recommended by NICE guidelines nevertheless polypharmacy is routinely prescribed in patients with BPD

**Objectives:**

To domenstrate the possibility of using Asenapine in treating Border line personality disorder

**Methods:**

Case :A case seen in our practice of a patient with Borderline Personality presenting with symptoms of affective instability,impulsivity,quasipsychotic symptoms that have not responded to trial of many different antipsychotics.She was started on Asenapine and experienced significant improvement in symptoms and daily functioning.However her medication was changed due to Asenapine being non formulary and this caused relapse in her mental state.She reported erratic sleep,poor appetite,anxiety and aggression.Asenapine was restarted and she improved.

**Results:**

Asenapine belongs to the chemical class of dibenzo-oxepino pyrroles and acts antagonistically at a number of receptors, and this combination of receptor-binding affinities differs from other available antipsychotics. Asenapine has high affinity for several 5-hydroxytryptamine (5-HT)-receptor subtypes, including 5- HT_2C_ , 5-HT_2A_ , 5-HT_7_ , 5-HT_2B_ , and 5-HT_6_(Musselman et al. AP 2021; 10.1177). Asenapine’s favourable weight and metabolic profile are of clinical interest. Asenapine was generally safe and well tolerated in paediatric patients (Dogterom et al. 2018 ; *Drug Des Devel Ther* 12:2677-2693). One open label study that looked at efficacy of Asenapine in BPD showed improvement with Asenapine in not only affective but also improve impulsive and cognitive symptoms( Martı´n-Blancoet al. ICP 2014 ;29(2):120-3). The results of both the CGI-BPD and the BSL-23 scales, which reflect the view of clinician and patients, respectively, show a significant improvement in the BPD general symptomatology ( Martı´n-Blancoet al. ICP 2014 ;29(2):120-3). In our case patient reported worsening of symptoms after Asenapine was discontinued.She experienced suicidal ideation, impulsivity, aggression, erratic sleep wake cycle and poor appetite.On restarting Asenapine there was significant improvement in her symptoms and marked subjective improvement in activities of daily living.

**Conclusions:**

Asenapine has therapeutic efficacy as well as good tolerability and safety profile.It can be used in patients with BPD especially when other antipsychotics have caused undesirable side effects like weight gain.

**Disclosure of Interest:**

None Declared

